# Therapeutic Interventions for *Pseudomonas* Infections in Cystic Fibrosis Patients: A Review of Phase IV Trials

**DOI:** 10.3390/jcm13216530

**Published:** 2024-10-30

**Authors:** Mohammed Alqasmi

**Affiliations:** Department of Medical Laboratory Sciences, College of Applied Medical Sciences, Shaqra University, Shaqra 11961, Saudi Arabia; malqasmi@su.edu.sa

**Keywords:** *Pseudomonas aeruginosa*, cystic fibrosis, chronic infection, antimicrobial resistance, clinical trials, phase IV

## Abstract

*Pseudomonas aeruginosa* (*Pa*) poses a significant threat to individuals with cystic fibrosis (CF), as this bacterium is highly adaptable and resistant to antibiotics. While early-stage *Pa* infections can often be eradicated with aggressive antibiotic therapy, chronic infections are nearly impossible to eliminate and require treatments that focus on long-term bacterial suppression. Without such suppression, these persistent infections can severely damage the lungs, leading to serious complications and a reduced life expectancy for CF patients. Evidence for a specific treatment regimen for managing Pa infections in CF patients remains limited. This narrative review provides a detailed analysis of antimicrobial therapies assessed in completed phase IV trials, focusing on their safety and efficacy, especially with prolonged use. Key antibiotics, including tobramycin, colistin, meropenem, aztreonam, ceftolozane/tazobactam, ciprofloxacin, and azithromycin, are discussed, emphasizing their use, side effects, and delivery methods. Inhaled antibiotics are preferred for their targeted action and minimal side effects, while systemic antibiotics offer potency but carry risks like nephrotoxicity. The review also explores emerging treatments, such as phage therapy and antibiofilm agents, which show promise in managing chronic infections. Nonetheless, further research is necessary to enhance the safety and effectiveness of existing therapies while investigating new approaches for better long-term outcomes.

## 1. Introduction

Cystic fibrosis (CF) is an incurable disease that affects the production of healthy mucus. This leads to a buildup of abnormal mucus that damages the lungs, digestive system, and other vital organs [1,2]. The global prevalence of diagnosed CF cases remains challenging to determine due to underdiagnosis limitations. However, the estimates suggest CF cases range from approximately 70,000 to over 160,000 [3,4]. The variations in CF prevalence across studies can be attributed to the heterogeneous approaches implemented by individual countries in their CF-screening programs [5]. This underscores the need for standardized methodologies to investigate CF prevalence on a global scale.

CF is caused by defects in the cystic fibrosis transmembrane conductance regulator (CFTR) gene, following an autosomal recessive pattern of inheritance. An individual inherits two copies of the CFTR gene, one from each parent; however, only those who receive two mutated copies will develop the disease. [6]. Despite the existence of over 2000 different genetic variations in the CFTR gene, around 700 are identified as disease-causing variants [7,8,9]. Moreover, data suggest that the prevalence of CFTR mutation carriers ranges from 4% to 5% in the general population [10,11,12,13].

In a healthy state, the CFTR protein resides in the apical membrane of epithelial cells in organs like the lungs, liver, pancreas, intestines, and sweat glands. It functions as a chloride channel, regulating the movement of ions and water across these cells, maintaining a thin low-viscosity layer of mucus [14,15]. However, in patients with CF (pwCF), the absence or malfunction of the CFTR protein leads to the production of thick, sticky mucus that can damage organs over time, leading to serious health complications [15].

Lungs are the organs most severely affected in pwCF due to impaired mucociliary clearance caused by thickened mucus secretions. This dysfunction traps inhaled debris and pathogens, creating a breeding ground for chronic infections that are often accompanied by a chronic neutrophilic inflammatory response [16,17]. While the inflammatory response is crucial for fighting infection, prolonged neutrophil recruitment and inflammation can contribute to collateral damage in the lung tissues [18]. Neutrophils exacerbate lung damage through destructive degranulation and the release of neutrophil extracellular traps (NETs), which consist of DNA and antimicrobial proteins. Although NETs are intended to trap and eliminate infectious agents, their dysregulation contributes to excessive inflammation and tissue destruction in CF patients. The prolonged presence of neutrophils and their harmful by-products further damages lung tissues, leading to a progressive decline in lung function and worsening disease severity [19].

*Pseudomonas aeruginosa* (*Pa*) poses one of the greatest concerns for lung infections in pwCF. While other bacterial threats like *Staphylococcus aureus* and *Haemophilus influenza* can also establish infections, particularly in the early stages of life, *Pa* is particularly dangerous due to its ability to thrive in damaged airways, contribute to progressive lung disease, and resist antibiotic treatment [20,21]. By adulthood, it is estimated that *Pa* colonizes the airways of up to 80% of CF individuals, significantly impacting their health [22,23,24]. It has been shown that in the chronic state, *Pa* isolates are predominantly mucoid, often overexpressing alginate, which contributes to the formation of highly structured biofilms [25,26]. Furthermore, within biofilms, a subpopulation of bacterial cells (approximately 1%) enters a dormant state known as persistence, which exhibit neither active growth nor immediate death, even when exposed to high concentrations of antibiotics [27].

The early detection of *Pseudomonas* infection is crucial for improving the life expectancy of pwCF, as eradication therapy with intensive antibiotics is most successful in newly diagnosed cases [28,29,30]. However, once chronic infection is established, *Pa* becomes resistant to eradication, leading to more severe complications. Chronic *Pa* infections significantly accelerate the decline in lung function in pwCF, with estimates suggesting a 5–10% decrease in lung capacity per year. This decline can lead to serious complications, including early death [31,32,33]. To address this, a multi-pronged strategy is typically employed to manage the infection burden and increase life expectancy. This involves antimicrobial treatment to suppress bacterial growth, anti-inflammatory medications to manage chronic lung inflammation, and airway clearance therapies, including CFTR modulators, which enhance chloride transport, improve mucus viscosity, and reduce the frequency and severity of lung infections by preventing bacterial build-up [34,35,36]. A recent study in the US reported a significant reduction in mortality rates, with the median age of death increasing from 24 years in 1999 to 37 years in 2020, a change attributed to the implementation of aggressive multi-approach treatments in pwCF [37].

## 2. Overview of Phase IV Clinical Trials

Phase IV clinical trials are conducted after a drug or therapy has received regulatory approval and becomes available to the public. These studies, which include post-marketing surveillance (PMS), are crucial for evaluating the long-term safety, effectiveness, and potential adverse effects of treatments in a broader and more diverse patient population than those involved in earlier trial phases [38,39]. Unlike phases I–III, which are performed under controlled conditions with carefully selected participants, phase IV trials examine how therapies perform in real-world settings, offering a deeper insight into their practical applications, potential risks, and broader efficacy (Table 1) [40].

Modifying an approved treatment plan to suit a specific population, such as managing infections in pwCF, qualifies as a phase IV trial [38,39]. For CF patients, who often require prolonged therapy, PMS is particularly important for monitoring the long-term safety and efficacy of treatments [41]. The real-world data collected through phase IV trials enable clinicians to determine whether these therapies maintain their effectiveness over time, reduce the frequency of exacerbations, and ensure an acceptable safety profile for prolonged use. Additionally, these trials can identify emerging issues such as drug resistance and individual variations in response, and allow for the refinement of treatment regimens to improve the long-term outcomes in CF care [42].

## 3. Therapeutic Candidates at Phase IV for Management of *Pa* Infection in pwCF

Despite the significant advancements in the management of infections in pwCF, ongoing research remains critical to further enhance both patient longevity and quality of life. The development of safer and more efficacious therapeutic strategies to address pseudomonal infections is paramount, given their persistent role in disease progression and associated complications [43]. This review analyzes antipseudomonal therapies assessed in completed Phase IV trials for the treatment of *Pa* infections in pwCF, providing valuable insights into the effectiveness and safety of current treatment modalities. Additionally, it aims to identify gaps in existing therapeutic approaches, offering a foundation for optimizing future strategies to better manage *Pa* infections in the CF population. Data for these clinical trials were sourced from the ClinicalTrials.gov database, which offers detailed information on ongoing and completed studies. A more in-depth discussion of these trials is provided in the sections below (Table 2).

### 3.1. Tobramycin

Tobramycin is a potent antibiotic that belongs to the aminoglycoside class. It demonstrates bactericidal activity against a broad spectrum of Gram-positive and Gram-negative bacteria including *Pa*, *Escherichia coli*, *Serratia*, *Enterobacter*, and *Klebsiella* spp. as well as both penicillinase-producing and non-producing strains of *S. aureus* [44,45]. Tobramycin works by interfering with protein synthesis machinery in bacteria. It binds to the 16S ribosomal subunit, an essential component of the bacterial ribosome responsible for translating mRNA into proteins. This binding disrupts the ability of bacteria to assemble functional proteins, ultimately resulting in cell death [46].

Following the U.S. Food and Drug Administration (FDA) approval in 1975, tobramycin has been established as a versatile therapeutic agent for managing and treating a diverse range of systemic and ocular infections [47,48,49]. It can be administered through various routes depending on the type of infection being treated, with available formulations for inhalation, injection, and topical ophthalmic application. However, the oral administration of tobramycin is limited due to poor bioavailability caused by efflux via P-glycoprotein pumps in the intestinal brush border [50,51,52].

Inhaled tobramycin has emerged as a key therapeutic tool to combat lung infections in pwCF as it is delivered directly to the airways via nebulization or dry powder inhalers. This localized approach minimizes systemic exposure and associated adverse effects (AEs) compared with intravenous antibiotics [50,53,54,55]. The AEs with long-term intake include nausea, vomiting, diarrhea, balance, and hearing problems and, most importantly, kidney damage with prolonged and/or higher doses [45,56,57]. 

### 3.2. Colistin

Colistin is a polycationic antibiotic belonging to a class of antibiotics called polymyxins. Colistin, also known as polymyxin E, was first isolated from the bacterium *Bacillus colistinus* in Japan during 1947. Notably, it exhibits broad-spectrum bactericidal activity against a diverse range of Gram-negative bacteria, especially those characterized by multidrug resistance (MDR) including *Enterobacteriaceae*, *Pa*, and *Acinetobacter baumannii* [58,59].

Colistin targets the lipopolysaccharides (LPS) structure in the outer membrane of Gram-negative bacteria for their antibacterial effect [58]. Bacterial LPS is made up of three main components: (i) a core oligosaccharide linked to the inner membrane; (ii) an outer polysaccharide chain (O-antigen); and (iii) a lipid A portion embedded in the outer membrane, maintaining its integrity. Hence, the electrostatic exchange between the positively charged Dab (diaminobutyric acid) residue on colistin and the phosphates of lipid A disrupts the bacterial membrane, which leads to cell death due to the escape of essential electrolytes and cellular components [58,60]. Additionally, colistin can also bind to the free molecules of LPS after bacterial lysis and neutralizing their toxic effects [61].

Although colistin received FDA approval in 1959, safety concerns about neurotoxicity and nephrotoxicity led to restrictions on its use in the early 1970s. However, more recently, it has re-emerged as a last-line therapeutic option for treating infections unresponsive to conventional antibiotics. This was driven by its superior effectiveness against MDR pathogens and the rarity of colistin resistance [62,63,64].

Colistin exists in two clinically relevant forms with distinct uses. Colistin sulfate (CS) is the active form and is suitable for oral, inhalation, or topical application. In contrast, colistin methanesulfonate sodium (CMS) is a prodrug that needs conversion in the body to become the active form of colistin. This form is intended for parenteral administration (injection or infusion) and may be given by inhalation. For long-term treatment plans, CMS is preferred because it is less toxic and has fewer unwanted effects than the active form of colistin [65,66,67]. The primary concern of colistin lies in its nephrotoxicity, which can lead to kidney damage. This risk increases with higher doses, longer treatment durations, and pre-existing kidney problems. Other reported side effects include neurotoxicity and gastrointestinal issues like nausea, vomiting, and diarrhea [62,68]. 

### 3.3. Meropenem

Meropenem is a carbapenem-class, broad-spectrum beta-lactam antibiotic that is highly effective against a wide variety of Gram-positive and Gram-negative bacterial infections [69]. It exerts a bactericidal effect by hindering the construction of the bacterial cell wall. This occurs when the meropenem binds to penicillin-binding proteins (PBPs), enzymes essential for cross-linking peptidoglycan strands in the cell wall. This binding disrupts the formation of a strong and stable cell wall, ultimately leading to the weakening and death of the bacteria [70,71]. Importantly, unlike its predecessor penicillin, meropenem is resistant to degradation by the chromosomal and extended-spectrum beta-lactamase enzymes produced by bacteria to deactivate beta-lactam antibiotics [72].

In response to growing concerns about antibiotic resistance in the 1990s, meropenem entered clinical use and quickly became a cornerstone in treating serious infections, including pulmonary infections in CF patients [73]. Meropenem comes in one form as a sterile powder for injection and it is typically administered intravenously (IV) by infusion method. This is because the orally administered formulations suffer from instability within the gut, poor permeability across the intestinal wall, and consequently, low bioavailability [74,75,76]. 

Meropenem is generally well-tolerated but can cause side effects. Common ones include injection site reactions, nausea, vomiting, and diarrhea [73]. Meropenem can also disrupt the gut flora, which can lead to metabolic shifts, and increases gut susceptibility to colonization by unwanted pathogens such as *Clostridioides difficile* [77,78].

### 3.4. Ceftolozane/Tazobactam

Ceftolozane/tazobactam (CZ/TAZ) is a synergistic antibiotic combination comprising a fifth-generation cephalosporin (ceftolozane) and a beta-lactamase inhibitor (tazobactam). This combination exerts its bactericidal effect against a wide range of organisms including *Pa* by inhibiting bacterial cell wall production while simultaneously preventing the beta-lactamase-mediated inactivation of ceftolozane [79,80]. 

CZ/TAZ has received recent approval by both the FDA and the European Medicines Agency (EMA) under the brand name Zerbaxa for treating certain complicated infections. The targeted infections include hospital-acquired pneumonia, complicated urinary tract infections, and intra-abdominal infections. CZ/TAZ is available in only one form as a sterile powder for injection which demonstrates versatility in treating complex infections. However, CZ/TAZ can be combined with metronidazole when anaerobic bacterial involvement is suspected [81,82,83].

Although CZ/TAZ is generally well-tolerated, it comes with certain safety considerations and potential interactions. Common side effects include injection site reactions, such as pain or irritation, as well as gastrointestinal symptoms like nausea, diarrhea, and headaches. While these are typically mild, more serious adverse effects, including muscle cramps, irregular heart rhythms, or kidney dysfunction, can occur in rare instances. Severe allergic reactions, such as anaphylaxis, are possible, particularly in patients with a history of allergies to cephalosporins, penicillin, or other beta-lactam antibiotics, making it crucial to evaluate any hypersensitivity before beginning treatment. Additionally, the prolonged use of CZ/TAZ may predispose patients to secondary infections, including oral thrush or yeast infections, and it carries a risk of developing a serious *C. difficile*-associated intestinal condition, characterized by persistent diarrhea and abdominal pain, which may arise during or after treatment [81,82].

### 3.5. Azithromycin

Azithromycin, a broad-spectrum macrolide antibiotic, is extensively used for treating respiratory infections and specific sexually transmitted infections (STIs). It is a derivative of erythromycin, with enhanced potency against Gram-negative bacteria, particularly *Enterobacteriaceae* and many Gram-positive organisms [84,85]. Azithromycin exerts its antibacterial effect by selectively binding to the 23S rRNA of the 50S ribosomal subunit in susceptible bacteria. This binding disrupts protein translation, thereby inhibiting bacterial growth and replication [86]. While primarily acting as a bacteriostatic agent, azithromycin can exhibit bactericidal effects at higher doses against certain bacteria like *H. influenzae* and a number of streptococci members [87,88].

Beyond its antibacterial role, azithromycin is recognized for its significant immunomodulatory effects, which are particularly beneficial in managing chronic respiratory diseases. These effects are mediated through multiple mechanisms, including the regulation of cellular signaling pathways and the suppression of pro-inflammatory cells and mediators [89,90]. Specifically, azithromycin suppresses the activation of nuclear factor kappa B (NF-κB), a key transcription factor involved in the production of inflammatory cytokines like IL-6, IL-8, and TNF-α [91,92]. In addition, it reduces the recruitment and activation of neutrophils and macrophages, thereby dampening excessive inflammatory responses [93,94]. Another key mechanism is its ability to inhibit the production of reactive oxygen species (ROS), which helps mitigate oxidative stress in the airways. Through these pathways, azithromycin not only helps control acute inflammation but also prevents the progression of chronic inflammation and subsequent tissue damage, ultimately improving lung function and reducing the frequency of exacerbations in conditions like cystic fibrosis and bronchiectasis [95,96].

Azithromycin can be administered by both the oral and parenteral routes. It has extended-release formulation coupled with comprehensive tissue and intracellular distribution, allowing once-daily dosing and a more concise treatment regimen compared with other antimicrobial agents [97,98]. Moreover, azithromycin is generally recognized as a well-tolerated antimicrobial agent, with a low incidence of discontinuation due to AEs. It also has a superior safety profile compared with other macrolides such as clarithromycin and erythromycin. Hence, it can be employed for long-term prophylaxis with reported potential for reducing exacerbation frequency, sputum volume, and improving lung function in non-cystic fibrosis bronchiectasis patients [99,100,101].

### 3.6. Ciprofloxacin

Ciprofloxacin belongs to the second-generation class of fluoroquinolone antibiotics. While it has broad-spectrum activity, it is particularly potent against Gram-negative organisms, including *Pa*, *E. coli*, and *Klebsiella pneumoniae* [102,103]. As a fluoroquinolone, ciprofloxacin functions as a bactericidal agent by inhibiting bacterial DNA gyrase and topoisomerase IV, which are crucial enzymes for bacterial DNA replication and repair. By disrupting these processes, it prevents the unwinding and re-ligation of bacterial DNA, leading to DNA fragmentation and, ultimately, bacterial cell death [104,105].

Ciprofloxacin can be administered orally or intravenously, making it a versatile agent for various clinical conditions. It is indicated for the treatment of multiple infections, including respiratory tract infections such as chronic bronchitis and bacterial pneumonia, as well as urinary tract infections (UTIs), including complicated cases like pyelonephritis, and gastrointestinal infections [106,107,108,109]. Oral formulations are available as oral suspensions and immediate- and extended-release tablets, while IV treatment is used for more severe or complicated infections where higher systemic concentrations are required or when oral administration is not feasible [110].

Ciprofloxacin is generally well-tolerated, but like all fluoroquinolones, it carries specific safety concerns. Common side effects include gastrointestinal disturbances (nausea, diarrhea), headaches, and dizziness. More serious, though less frequent, adverse effects include tendonitis, QT prolongation, and central nervous system (CNS) effects such as seizures, particularly in elderly patients or those with underlying conditions [111,112,113]. However, ciprofloxacin is contraindicated in pediatric patients and pregnant women due to concerns over arthropathy [114,115].

### 3.7. Aztreonam

Aztreonam is a monobactam antibiotic, part of the beta-lactam family, which is distinguished by its unique single-ring structure. Unlike other beta-lactams, such as penicillin and cephalosporins, its structure allows it to target a narrow range of bacteria. Aztreonam is highly effective against Gram-negative bacteria, including *Pa*, *E. coli*, *K. pneumoniae*, and *Enterobacter* species. However, it has little to no activity against Gram-positive bacteria or anaerobes, making it a specialized treatment option for Gram-negative infections [116,117].

Aztreonam is indicated for the treatment of a range of infections, including respiratory infections such as pneumonia and bronchiectasis, UTIs, septicemia, skin and soft tissue infections, as well as intra-abdominal and gynecological infections caused by Gram-negative bacteria [118,119,120]. It is typically administered either IV or intramuscularly (IM) for systemic infections, as it is poorly absorbed when taken orally [116]. However, for chronic lung infections, aztreonam is also available in an inhaled form, known as aztreonam lysine (AZLI) [119]. 

Known for its generally favorable safety profile, aztreonam is well-tolerated by most patients. Common side effects include mild reactions at the injection site, gastrointestinal issues like nausea, vomiting, and diarrhea, and occasional rashes. More serious side effects, such as allergic reactions and elevated liver enzymes, are rare [121,122]. A major advantage of aztreonam is its safe use in individuals with allergies to other beta-lactam antibiotics, such as penicillin and cephalosporins, as cross-reactivity is exceptionally uncommon [116].

## 4. Treatment Approaches for Management of Pa Infections in pwCF

Antibiotics are a key component of a multifaceted approach to manage infections in pwCF which includes anti-inflammatory therapies, mucolytics, bronchodilators, and CFTR modulators to improve lung function and reduce mucus build-up [36,123]. Around 80% of newly acquired *Pseudomonas* infections can be eradicated using a combinatorial therapeutic approach involving oral, inhaled, and intravenous antibiotics [124]. However, once the infection transitions to a chronic state, eradication becomes nearly impossible, and treatment focuses primarily on suppressing bacterial load and managing symptoms. The optimal antibiotic regimens, including the best combination of drugs, appropriate dosages, and treatment durations, are still areas of active investigation [125,126,127,128]. To date, no single antibiotic or established combination has been proven to achieve the definitive eradication of *Pa* in pwCF [129]. The diversity of these approaches reflects the ongoing search for the most effective treatment strategies for this complex and long-lasting condition.

### 4.1. Systemic Drugs

Systemic antibiotics are often chosen for their ability to achieve high systemic concentrations, making them effective in treating severe or widespread infections [130]. However, while systemic antibiotics can be highly effective in the short term, their long-term use raises concerns, including their potential toxicity and alterations in the pathogenicity of gut microbiota [131,132]. Limited data exist regarding the safety profiles of intravenous (IV) tobramycin and colistin for treating acute pulmonary exacerbations (PEx) in CF patients. Concerningly, IV tobramycin has demonstrated a significantly higher frequency of AEs compared with the two IV-dosing regimens of colistin (NCT02918409). Nephrotoxicity emerged as the most significant risk associated with IV tobramycin treatment, potentially leading to treatment failure and a worsening of the patient’s condition. This was evidenced by a higher incidence in patients receiving IV tobramycin (25%) compared with only 4% in those treated with both colistin regimens combined in this two-week duration study. In fact, nephrotoxicity is a well-recognized AE associated with IV administration of anti-*Pseudomonas* medications like tobramycin and colistin, as observed in several previous studies [133,134,135,136]. Notably, it has been demonstrated that renal function can recover to pre-treatment levels within 4 weeks after discontinuing tobramycin [137]. In contrast, colistin-induced nephrotoxicity exhibits a dose- and time-dependent relationship, with reported incidence varying between 10% and 70% across multiple studies [138,139,140,141,142]. This variation can also be attributed to the different diagnostic criteria and targeted groups of patients employed by studies [143]. Thus, the systemic administration of antibiotics presents a double-edged sword, necessitating judicious use, especially for chronic conditions. 

### 4.2. Inhaled Drugs

Studies have shown that inhaled antibiotics are successful in reducing *Pa* density in the airways, improving lung function, and decreasing the frequency of PEx in pwCF with chronic infections [55,144]. Inhaled antibiotics offer a distinct advantage over systemic treatment because of their reduced systemic toxicity and the ability to achieve higher concentrations within the respiratory tract. Despite that, mild to serious AEs can arise in most patients with prolonged use [45,49,145]. In an extended phase IV study focused on the long-term safety of tobramycin inhalation powder (TIP) (NCT01519661 and NCT01775137), the drug demonstrated good tolerability with no unpredictable safety signals identified, and its efficacy was maintained throughout the observation period [146]. The reported AEs were comparable to those observed in other long-term studies from phase III [146,147,148]. Infective PEx was the most common adverse event and the primary reason for study discontinuation with a comparable severity to those in relevant studies [146,147,148]. Cough, the second most common adverse event, was found to decrease in frequency over time. This differentiates this phase IV investigation from previous trials that reported consistently higher rates of cough [146,147,148]. This decrease in cough frequency may be attributed to the improved inhalation techniques by patients, and the effectiveness of TIP in rapidly reducing bacterial load over time. This reduction in bacteria likely leads to a subsequent decrease in neutrophilic airway inflammation, which in turn, reduces sputum production and purulence [149,150,151]. While hemoptysis was also common, most cases were mild and likely linked to the underlying disease, as explained earlier by Thompson et al. (2015) [146,152]. Overall, TIP can be considered a safe and tolerable long-term treatment option for CF patients with established lung disease, although some AEs warrant monitoring. However, limitations inherent to the phase IV open-label, single-arm design necessitate consideration [146]. This is because the design is susceptible to reporting bias, especially for subjective AEs. Furthermore, the lack of a comparator group restricts the ability to definitively assess the treatment’s relative efficacy and safety profile when compared with alternative therapies or placebo. 

Following the 1980s’ success of localized delivery of tobramycin, more effective treatments for chronic *Pa* infections in CF have been introduced via inhalation including colistin, aztreonam, and fluoroquinolones [153]. Nebulizers and dry powder inhalers (DPIs) represent two primary modes of direct drug delivery to the respiratory tract. In a comparative study between TIP and nebulized solutions of tobramycin (TIS) and colistimethate (NCT01844778), TIP administration via the T-326 inhaler demonstrated improved usability, reduced total delivery time, and significantly lower contamination rates compared with nebulizers [154]. Indeed, several studies have demonstrated that the reduced administration time and user-friendliness of inhaler devices positively impacts patient adherence to treatment regimens. This ease of use likely translates to minimized medication-handling errors and improved patient satisfaction with the therapy [155,156,157,158,159]. Moreover, DPIs have minimal risk of microbial contamination as frequent cleaning between uses is unnecessary [160]. In spite of the advantages, DPIs have a number of limitations compared with nebulizers. Certain antibiotics are currently unavailable in DPI formulations, restricting their use in this delivery method. Furthermore, the effective utilization of DPIs often requires a proper inhalation technique, potentially limiting their suitability for young children, older adults, or patients with compromised conditions [161]. 

### 4.3. Combination Therapies

While combination therapies offer the potential advantage of broader coverage against resistant bacteria, a careful evaluation of potential side effects and drug interactions is needed [162]. A common regimen involves TIP with oral azithromycin, a treatment prescribed to approximately 75% of patients with chronic lung infections [163]. However, the efficacy of this combination remains unclear. In a phase IV trial intended to investigate the impact of adding azithromycin to a standard TIP regimen (NCT02677701), the co-administration of azithromycin with TIP did not produce a synergistic effect in terms of improving lung function, nor did it result in significant changes in AEs or bacterial load when compared with a placebo [164]. Similarly, Mayer-Hamblett et al. (2018) reported no significant difference in the eradication rate of early *Pa* infection or clinical outcomes between CF patients receiving tobramycin solution for inhalation (TIS) with azithromycin and those receiving TIS alone [165]. On the other hand, several randomized controlled trials involving azithromycin have demonstrated improvements in lung function, a decrease in PEx and hospitalizations, and a reduced need for intravenous antibiotics in pwCF [166,167,168]. These benefits are likely attributable to the anti-inflammatory properties of azithromycin when used long-term. This can be explained by the shorter duration of a phase IV study (NCT02677701) compared with the other controlled trials and also that the Mayer-Hamblett study (2018) targeted early-stage infections before the establishment of chronic *Pa* colonization [165]. Therefore, the long-term use of azithromycin could be beneficial in improving lung function as a prophylactic measure, not through direct antimicrobial activity, but by mitigating inflammatory consequences and disease progression. However, more optimized studies are required to confirm these benefits in CF patients.

### 4.4. Optimal Dosing

Emerging evidence suggests that optimal dosing regimens may differ in CF population compared with non-CF patients [169]. Medication misuse, including overuse, can lead to undesirable side effects, compromising the intended therapeutic benefit and potentially culminating in treatment failure. To address this, two studies (NCT01429259 and NCT02421120) investigated the pharmacokinetics and tolerability of meropenem and ceftolozane/tazobactam (CZ/TAZ) in children and adult CF patients experiencing acute PEx, respectively. Interestingly, meropenem exhibited a higher clearance rate compared with previously reported values in healthy children [170,171,172,173]. In contrast, the findings of CZ/TAZ clearance revealed no significant difference between adult with and without CF condition [174,175,176]. However, the conclusive determination of optimal dosing regimens remains uncertain due to limitations in both phase IV studies. The relatively small sample sizes and the comparable baseline characteristics of the patient groups limit the generalizability of the findings. Larger and well-designed studies are warranted to definitively establish whether dosing strategies for these antibiotics differ between CF and non-CF populations.

### 4.5. CFTR Modulators

CFTR modulators have been shown to reduce *Pa* load in the sputum of CF patients [177,178]. These modulators include potentiators, such as Ivacaftor, which maintain the CFTR channels in an open state, allowing chloride ions to flow across cell membranes, and correctors, like Lumacaftor, which help ensure the protein folds correctly and reaches the cell surface [179,180,181]. Although they do not act as antibiotics, restoring CFTR function improves the lung environment, enhancing the effectiveness of antibiotic treatments by improving their penetration into biofilms and reducing the risk of reinfection by facilitating mucus clearance and decreasing bacterial colonization [177]. However, research in adults has shown that while *Pa* load decreases, chronic infections often persist and remain difficult to fully eradicate, particularly in individuals with advanced lung disease [178].

## 5. Challenges and Limitations in Treatments

*Pa* infections remain a major challenge in the management of CF patients, complicating efforts to improve the outcomes in this incurable condition. The genetic and phenotypic diversity of *Pa* populations in pwCF plays a critical role in treatment challenges and failures. This diversity allows bacterial subpopulations to transition between acute and chronic infection states, driven not only by mutations and the transfer of mobile genetic elements (MGEs) but also by the bacterium’s ability to modulate gene expression in response to environmental cues within the CF lung [182,183,184,185]. This on/off genetic regulation enables *Pa* to adapt dynamically to fluctuating conditions such as oxygen levels, nutrient availability, and immune pressures. Such adaptability promotes biofilm formation, antibiotic resistance, and immune evasion, allowing the pathogen to persist and thrive in the hostile lung environment. These adaptive strategies are particularly problematic in the context of the underlying pathophysiological conditions in CF patients, where thickened mucus and compromised immune responses create an ideal environment for *Pa* colonization [186,187,188]. The combined effects of genetic variability, biofilm development, and phenotypic flexibility further complicate efforts to eradicate the bacterium, often leading to persistent and recurrent infections despite aggressive therapy. This underscores the urgent need for continuous monitoring, personalized treatment strategies, and the reassessment of current therapeutic approaches to ensure effective long-term management of Pa infections in CF care [23,189,190,191,192].

*Pa* can readily develop resistance to most antibiotics. This situation is further complicated by the fact that prolong antibiotic exposure in pwCF selects for aggressive resistant strains, which makes it increasingly difficult to find effective antibiotics for treatment [193]. *Pa* is also notorious for its ability to form complex structures called biofilms. These complex structures can significantly impede therapeutic efforts by creating a formidable physical barrier around bacterial cell, effectively evading both immune defences and antibiotic action. Bacteria residing within biofilms also exhibit a downregulated metabolic state, leading to a restricted influx of external substrates [194,195]. It has been found that persister cells within the biofilm can tolerate up to 1000× minimum inhibitory concentration (MIC) of antibiotics under in vitro conditions [27,196].

The complex interplay between abnormal mucus production, impaired mucociliary clearance, and biofilm formation by *Pa* creates a fertile ground for chronic and recalcitrant infections in CF patients which can trigger the development of severe symptoms [197]. The presence of *Pa* infection is associated with more than 2.5-fold increase in the risk of mortality in CF patients over an 8-year period [198,199]. Acute PEx represents the worsening of symptoms and most common cause of death in CF patients. In fact, the increased frequency of PEx contributes to accelerated lung function decline and diminished quality of life [200,201]. As shown by two independent prognostic models, the higher frequency of annual cystic fibrosis PEx was directly linked to decreased 2-year and 5-year survival rates [202,203]. However, the prompt detection of bacterial cases accompanied by multifaceted management are crucial to minimize the risk, severity, and duration of exacerbations [204].

## 6. Emerging Treatments

Beyond conventional options, promising emerging therapies are demonstrating potential in the fight against *P. aeruginosa* infections in CF. Phage therapy is considered to be the most promising approach to treat CF infection [205]. Phages (or bacteriophages) are bacterial viruses with a remarkable degree of host specificity, exclusively targeting and lysing bacterial cells without affecting human cells. They were used as a therapeutic option in the early 20th century, particularly in Eastern Europe, before the widespread discovery and adoption of antibiotics. However, since the challenge of antimicrobial resistance intensifies, phage therapy re-emerges as a promising strategy for managing infections caused by MDR bacteria [206,207].

Gene-editing methods offer significant potential for addressing microbial infections in pwCF, either by fixing the CFTR gene mutation, which is the root cause of complications, or by directly targeting the causative pathogens [208,209,210,211]. CRISPR-Cas technology has emerged as a leading tool in this area. It can be customized to specifically target a CFTR mutation using a guide RNA (gRNA), which directs the Cas enzyme to the defective DNA sequence, enabling correction of the CFTR mutation. This restoration of normal CFTR function improves mucus clearance and reduces the likelihood of chronic infections [209,210]. Furthermore, CRISPR-Cas can be adapted to directly fight *Pa* infections by targeting bacterial DNA. This can be achieved by designing gRNAs that bind to specific genes responsible for bacterial virulence or antibiotic resistance, leading to the disruption of these genetic elements. As a result, the bacteria become less able to form biofilms or resist antibiotics, making it easier for the immune system to clear the infection [208,211]. 

Additionally, the development of biofilm-disrupting agents offers a potential solution against chronic bacterial infections [212]. These agents target the structural integrity of the biofilm matrix, which is composed of extracellular polymeric substances (EPS) that shield bacteria from antibiotics and the immune system. These agents can degrade the EPS, inhibit its formation, or disrupt quorum sensing that regulate biofilm development. By breaking down this protective barrier, the bacterial cells become more susceptible to antibiotic treatment and immune clearance [213]. One recent example is the human hormone hANP which has demonstrated promise in dismantling *Pa* biofilms and enhancing the efficacy of conventional antibiotics [214]. However, large-scale clinical trials are necessary to validate the efficacy and safety of these emerging therapies, especially in the context of CF. Additionally, addressing the potential for phage resistance and ensuring cost-effectiveness of these novel approaches requires further investigation.

## 7. Conclusions

*P. aeruginosa* infections continue to pose a significant challenge in CF management. While current antibiotic therapies form the foundation of treatment, their limitations necessitate the exploration of alternative and complementary strategies. Phase IV trials play a crucial role in optimizing current regimens, and promising options like phage therapy and gene editing offer hope for the future. By adopting a multifaceted approach that incorporates these advancements alongside conventional methods, we can achieve an effective control for *Pa* infection and improve the quality of life for CF patients.

## Figures and Tables

**Table 1:** Clinical Trial Phases.

Pre-Clinical	Phase I	Phase II	Phase III	Phase IV
Involves laboratory and animal studies to evaluate the safety, biological effects, and therapeutic potential of a new drug or treatment. *Aims* Identifies possible toxicities and pharmacokinetic profiles. Determines if a drug is suitable for human trials.	The initial phase of human testing. Typically involves 10–80 healthy volunteers. *Aims* Assess the safety of a new drug. Establish safe dosage levels. Observe any early signs of adverse effects.	Includes a larger group of patients (100–300) with the condition the drug aims to treat. *Aims* Evaluates the drug’s effectiveness. Refines the dosing regimen. Continues to assess its safety profile.	Conducted with larger patient populations (1000–3000+). *Aims* Verifies the treatment’s efficacy. Monitor for side effects. Compares the new drug to existing standard therapies. Provides comprehensive data for regulatory approval.	Started after the drug approval and is available to the public. *Aims* Focuses on long-term safety, effectiveness. Identifies any rare side effects that may emerge with widespread use of a drug. Ensures the drug’s continued safety in a diverse patient population.

**Table 2:** Phase IV trials for the treatment of *Pa* infections in pwCF.

No	Study Title	Accession Number	Treatments	*N*	Start Date	Completion Date	Primary Measures (Endpoint)	Main Findings	Time Frame
1	Long Term Safety of Tobramycin Inhalation Powder in Patients with Cystic Fibrosis	NCT01519661 PMID(27435882)	Tobramycin inhalation powder (TIP)	157	01-2012	01-2014	The proportion of participants experiencing treatment-emergent adverse events (AEs), serious adverse events (SAEs), and deaths.	No new safety concerns emerged, and the intervention maintained its effectiveness throughout the study period	337 days
2	Ext. Long-term Safety Study in CF Patients: Single Arm TIP ^1^	NCT01775137	Tobramycin inhalation powder (TIP)	45	02-2013	11-2014	To determine the number of participants with AEs, SAEs, and AEs/SAEs leading to discontinuation of the study drug, and deaths across 12 treatment cycles.	The adverse event profile was similar to the core study.	673 days
3	IV Colistin for Pulmonary Exacerbations: Improving Safety and Efficacy	NCT02918409	Colistin Tobramycin	51	08-2016	11-2021	To evaluate the efficacy and safety of standard colistin dosing (2.5 mg/kg/day, administered three times daily, or TID) compared with a pharmacokinetically (PK)-adjusted colistin regimen administered twice daily (BID) at 5 mg/kg/day in adult cystic fibrosis (CF) patients with acute pulmonary exacerbation (APE). To assess the efficacy and safety of PK-adjusted BID colistin dosing compared with standard once-daily tobramycin dosing (8–10 mg/kg) in adult CF patients with APE.	Notable safety concerns were observed. IV tobramycin resulted in more renal toxicity events compared with other study arms. Both tobramycin and colistin resulted in an improvement in lung function.	Up to 14 days
4	Ease of Use and Microbial Contamination of Tobramycin Inhalation Powder (TIP) Versus Nebulised Tobramycin Inhalation Solution (TIS) and Nebulised Colistimethate (COLI)	NCT01844778 PMID(28614995)	Tobramycin inhalation powder (TIP) Tobramycin inhalation solution (TIS) Colistimethate	60	08-2013	10-2015	To evaluate the mean administration time of TIP using the T-326 inhaler compared with the total administration time of COLI or TIS.	The T-326 inhaler for TIP administration demonstrated ease of use, reduced total delivery time, and significantly lowered contamination rates compared with nebulizers.	Days 1, 28, 57, 84, 112
5	Population Pharmacokinetics of Prolonged Infusion Meropenem in Cystic Fibrosis (CF) Children	NCT01429259 PMID(26416780)	Meropenem	30	02-2012	01-2014	To determine total body clearance of meropenem at different concentrations by analyzing the pharmacokinetics of the study population. To determine the central compartment volume of meropenem at different concentrations by analyzing the pharmacokinetics of the study population.	Cystic fibrosis (CF) children exhibited higher meropenem clearance compared with previously reported values in non-CF children. Prolonged infusion improved drug exposure against pathogens with minimum inhibitory concentrations (MICs) ≥ 1 mg/L.	8-h dosing interval after 3rd meropenem dose /14–21 days
6	Population Pharmacokinetics and Safety of Intravenous Ceftolozane/Tazobactam in Adult Cystic Fibrosis Patients	NCT02421120 PMID(27550351)	Ceftolozane/Tazobactam	21	09-2015	10-2016	To evaluate the clearance of ceftolozane and tazobactam during an 8 h dosing interval. To assess the volume of distribution of ceftolozane and tazobactam over the same 8 h dosing interval.	Ceftolozane-tazobactam demonstrated good tolerability in this study. Ceftolozane and tazobactam clearance in cystic fibrosis (CF) patients was comparable to non-CF adult populations. AEs associated with ceftolozane-tazobactam were mild and potentially attributable to co-administered medications in most cases.	0, 1–1.08, 1.25–1.5, 2–3, 4–5, and 7–8 h after start of final dose/3 days
7	Testing the Effect of Adding Chronic Oral Azithromycin to Inhaled Tobramycin in People with Cystic Fibrosis (CF)	NCT02677701	Azithromycin plus inhaled tobramycin	119	10-2016	02-2020	To assess the effects of long-term oral azithromycin combined with inhaled tobramycin in adolescents and adults with CF and chronic *Pa* infections	No significant improvements in lung function were detected with the addition of Azithromycin.	6 weeks
8	Scandinavian Cystic Fibrosis Azithromycin Study	NCT00411736	Azithromycin plus inhaled colistin and ciprofloxacin	45	05-2008	03-2014	To assess if adding low-dose azithromycin to standard inhaled colistin and oral ciprofloxacin for treating intermittent *Pseudomonas* airway infections can delay recurrence and prevent progression to chronic airway infection.	No results reported.	Up to 5 Years
9	Comparison of 2 Treatment Regimens for Eradication of *P Aeruginosa* Infection in Children with Cystic Fibrosis	NCT01400750	Tobramycin inhalation solution (TIS) Inhaled colistimethate sodium plus oral ciprofloxacin (CC)	61	08-2001	05-2011	To compare the ability of two regimens to successfully achieve *Pa* eradication at the end of their treatment plans.	No results reported.	1 months for TIS 3 months for CC
10	Aztreonam for Inhalation Solution (AZLI) for the Treatment of Exacerbations of Cystic Fibrosis	NCT02894684	Aztreonam inhalation solution (AZLI)	16	01-2017	09-2019	To assess the clinical efficacy of AZLI in treating acute pulmonary exacerbations.	No results reported.	14 days

The data were retrieved on 27 September 2024 from ClinicalTrials.gov using the search terms “Cystic fibrosis” and “*Pseudomonas* infection”. Only completed phase IV trials were included. ^1^ An extension of study 1 at this table.
